# Time trends in nutrient intake and dietary patterns among five birth cohorts of 70-year-olds examined 1971–2016: results from the Gothenburg H70 birth cohort studies, Sweden

**DOI:** 10.1186/s12937-019-0493-8

**Published:** 2019-11-06

**Authors:** Jessica Samuelsson, Elisabet Rothenberg, Lauren Lissner, Gabriele Eiben, Anna Zettergren, Ingmar Skoog

**Affiliations:** 1 Neuropsychiatric Epidemiology Unit, Department of Psychiatry and Neurochemistry, Institute of Neuroscience and Physiology, Sahlgrenska Academy, Centre for Ageing and Health (AgeCap) at the University of Gothenburg, Gothenburg, Sweden; 2 Food and Meal Science, Kristianstad University, Kristianstad, Sweden; 3 Department of Community Medicine and Public Health, at the University of Gothenburg, Gothenburg, Sweden; 4 Department of Biomedicine and Public Health, University of Skövde, Skövde, Sweden

**Keywords:** Dietary patterns, Macronutrients, Micronutrients, Energy intake, Nutrient intake, Older adults, Diet history, Time trends

## Abstract

**Background:**

Nutrition is a key factor in healthy ageing but there are still gaps in knowledge about risk- and protective factors linking diet and healthy ageing. The aim of this study was to investigate time trends in dietary patterns and nutrient intake in an older population, in order to increase the understanding of whether dietary recommendations are followed and if nutrient needs are met.

**Methods:**

Cross-sectional data was derived from five samples of 70-year-olds examined 1971–72, 1981–83, 1992–93, 2000–02 and 2014–16 from the Gothenburg H70 birth cohort studies in Sweden. A total of 2246 individuals (56% women) participated. Dietary intake was determined by the diet history method, which is an interview including questions on usual frequencies and portion sizes of food intake during the preceding three months. Recommended values of nutrient intake and determinants of healthful dietary patterns were based on the Nordic Nutrition Recommendations 2012. Statistical analyses were performed using general linear models, student’s t-test and chi-square test, stratified by sex.

**Results:**

The intake of fruits and vegetables, fish and seafood, whole grain products and nuts and seeds increased during the study period (*p* < 0.0001), among both sexes. However, there was also an increase in alcohol intake (*p* < 0.0001), especially from wine and beer, and in 2014–16 more than 30% had an alcohol intake above recommendations. Protein intake increased (*p* < 0.0001 for women and *p* = 0.0004 for men), and 48% of the women and 37% of the men had a protein intake above recommended 1.2 g/kg body weight and day in 2014–16. The proportion of participants at risk of inadequate intake of vitamins C, D and folate decreased during the study period, among both sexes (p < 0.0001). However, vitamin D intake from diet was still below average requirement level of 7.5 μg/day for 49% of the women and 32% of the men in 2014–16.

**Conclusions:**

Dietary patterns have changed among 70-year-olds during the past five decades, with an increase in healthful foods and a higher nutrient density in later born birth cohorts. However, the intake of alcohol increased, especially among women. Results from this study can be useful as a basis for dietary guidelines and used for prevention strategies involving older adults in population-based and health care settings.

## Background

The average life expectancy is increasing world-wide and older adults constitute a growing proportion of the global population [1–3]. In Sweden, 20% of the population is ≥65 years of age, and by 2050 one in six worldwide, and one in four persons living in Europe and Northern America, is expected to be ≥65. Non-communicable diseases (NCDs) account for most of the disease burden in older adults over 60 years of age (about 85%), where cardiovascular diseases, cancer, diabetes and dementia account for approximately 50% [4]. Many of these conditions can be prevented or delayed with a healthy lifestyle [5–9].

Healthy ageing is the process of developing and maintaining the functional ability that enables wellbeing in older age (World Health Organization definition) [10]. Nutrition is a key factor in healthy ageing [9, 11, 12], but there are still gaps in knowledge about dietary patterns and nutrient intake among older adults, and whether intake is optimal in relation to recommendations in order to promote health and reduce the risk of NCDs [8, 13, 14]. Risk- and protective factors linking diet and healthy ageing can be identified by studying time trends of dietary intake in older populations, but these types of studies are rare. The Gothenburg H70 birth cohort studies in Sweden, have studied older adults since 1971 [15]. Previous results, up to 2000, indicate that later born birth cohorts have generally healthier dietary patterns, and that diet quality varies with level of education and occupation [16, 17]. A new birth cohort of 70-year-olds was added to the study in 2014 [18], which provides an opportunity to investigate time trends in dietary patterns and energy- and nutrient intake over nearly half a century in community-dwelling older adults.

The Nordic nutrition recommendations (NNR 2012) are developed to promote health and reduce the incidence of non-communicable diseases (NCD) [8]. The NNR 2012 are in line with global guidelines [8, 19–21], and form the basis for national dietary recommendations in the Nordic countries. These guidelines suggest that intake of foods, such as fruits and vegetables should be at least 500 g/day, that nuts and seeds and whole grain products should increase, and that fish and shellfish should be consumed 2–3 times a week. Further, they suggest that dairy products should be low in fat, and that intake of red and processed meat should be less than 500 g/week. They also suggest that alcohol intake should be less than 10 g/day for women and less than 20 g/day for men, and that salt and products high in added sugar should decrease in the population [8]. Comparing dietary intake among older adults with reference values from the NNR 2012 can increase our understanding of whether dietary recommendations are followed and if nutrient needs are met.

The primary aim of this study was to describe time trends in dietary patterns and energy- and nutrient intake in five population-based samples of 70-year-olds in the Gothenburg H70 birth cohort study, examined between 1971 and 2016. A secondary aim was to relate the results to the NNR 2012.

## Methods

### Samples

Data was derived from the Gothenburg H70 Birth Cohort Studies and the Population Study of Women in Gothenburg (PPSW) [15, 18, 22–24]. The H70 study, which started in 1971, is a multidisciplinary epidemiological study examining a representative sample of older individuals, systematically recruited via the Swedish Population Register (Statistics Sweden). The studies include detailed personal examinations, assessing a large number of age-related risk and protective factors associated with the normal ageing processes [18].

**Fig. 1:**
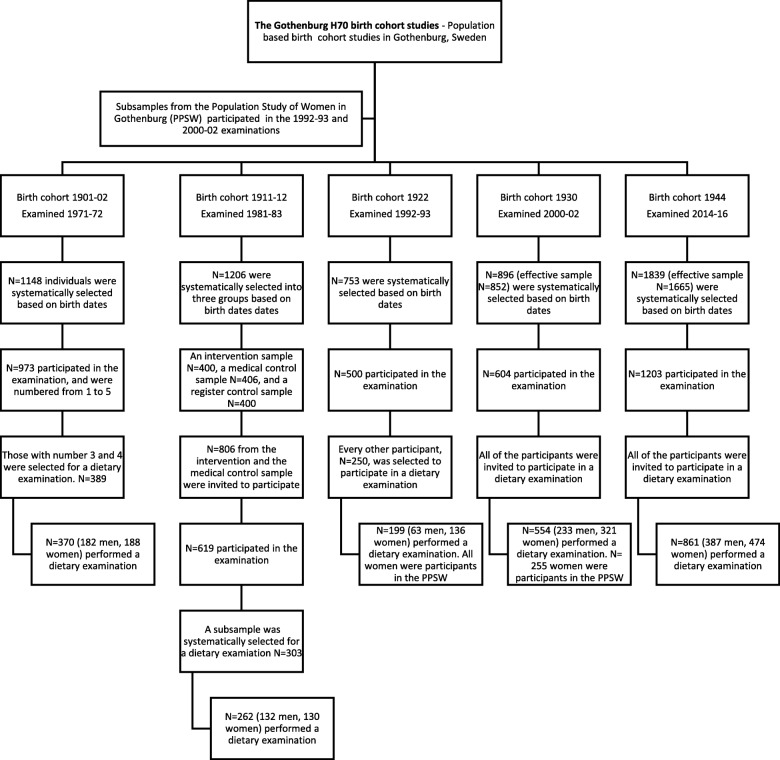
Diagram flow of study design

This study has a serial cross-sectional design. It includes 70-year-olds who completed the dietary part of the examination performed in 1971–72 (birth cohort 1901–02), 1981–83 (birth cohort 1911–12), 1992–93 (birth cohort 1922), 2000–02 (birth cohort 1930) and 2014–16 (birth cohort 1944), as described in Fig. 1. Inability to remember and communicate dietary intake was exclusion criteria. The birth cohorts and characteristics of participants and non-participants have been described in detail previously [16, 18, 25, 26].

#### Birth cohort 1901–02

In 1971–72, all 70-year-olds living in Gothenburg and born 1901 and 1902 between July 1st, 1901 and June 30th, 1902, were systematically selected based on birthdates ending with 2, 5 or 8 (*n* = 1148), as described previously [15, 25]. Among these, 973 (response rate 85%) participated in the examination, and were numbered from 1 to 5. Those with number 3 and 4 were selected for a dietary examination (*n* = 389). Among those, 370 (182 men, 188 women) performed a dietary examination, which took place in the participants’ home.

#### Birth cohort 1911–12

In 1981–83, a population sample of 70-year-olds (*n* = 1206) was systematically selected based in birth dates, as described previously [27]. The intervention concerned social, psychological, environmental and medical aspects in 400 participants, medical aspects alone in 406 participants and register control samples for 400 participants. A total of 619 (from the intervention and medical sample) participated in the examinations (response rate 77%). A subsample was systematically selected for the dietary examination (*n* = 303) [25]. Among those, 262 (132 men, 130 women) performed a dietary examination, which took place in the participants’ home.

#### Birth cohort 1922

In 1992–93, all 70-year-olds living in Gothenburg and born 1922 on days 6, 12, 18, 24, 30 of each month were invited to participate (*n* = 753), as described previously [24, 25, 28]. Among these, 500 participated (response rate 66%) in the examination. A subsample of 250 were selected for a dietary examination. Among those, 199 (63 men, 136 women) participated in the dietary examination, which was performed in an outpatient hospital department, as part of the basic H70 examinations. The examination was a cooperation between the PPSW in Gothenburg and the H70-study. Thus, all of the women had previously taken part in the longitudinal PPSW.

#### Birth cohort 1930

In 2000–02, all 70-year-olds living in Gothenburg and born 1930 on days 6, 12, 18, 24, 30 of each month were invited to participate (*n* = 896, effective sample *n* = 852), as previously described [16, 24, 26, 28]. A total of 604 (71%) participated in the examination. All participants were scheduled for the dietary examination. Among those, 554 (233 men, 321 women) participated in the dietary examination, which was performed in an outpatient hospital department, as part of the basic H70 examinations. The examination was a cooperation between the PPSW in Gothenburg and the H70-study. Thus, 255 of the women had previously taken part in the longitudinal PPSW.

#### Birth cohort 1944

In the 2014–16, all 70-year-olds living in Gothenburg and born 1944 on dates ending with 0, 2, 5, or 8 were invited to participate (*n* = 1839, effective sample *n* = 1665), as previously described [18]. A total of 1203 (72%) participated in the examination. All participants were invited to take part of the dietary examination. Among those, 861 (387 men, 474 women) participated in the dietary examination, which was performed either at the participants home or in an outpatient hospital department (the participants could choose).

### Data collection

#### Dietary examination procedure

The diet history (DH) method was used in all five birth cohorts [16–18, 25, 29], and results were therefore comparable. This DH has been validated and found to give estimated energy intakes comparable with energy requirements predicted by the heart rate method, activity diary and double labelled water method. However, with the double labelled water method, it was reported that the DH underestimated usual energy intake by 12%. The underreporting of energy intake was mainly observed among overweight participants [30, 31].

The dietary examinations were semi-structured face-to-face interviews, estimating dietary intake during the preceding three months. The protocols for the interviews consist of a meal-pattern interview, accompanied by a food list with questions on usual frequencies and portion sizes of foods. Dietary intake in the 2014–16 examination was registered as gram of food items usually consumed per day/week/month in a customized (for this study) version of the computer program Dietist Net [32] containing the Swedish National Food Agency’s (NFA) nutrient database of 2015. Pictures of foods, containing portion sizes, from NFA were used during the interviews. The previous examinations were registered in the same way and calculated in the NFA’s nutrient database (PC-kost) in 2000–02 [16]. The dietary examinations have been described in detail previously [16, 18, 25]. Trained dietitians performed the interviews in all of the five birth cohorts.

#### Dietary assessments

To evaluate potential underreporting of energy intake in this study, the Goldberg method was used [33]. Energy intake (EI) in megajoul (MJ) was divided with calculated basal metabolic rate (BMR) values [33]. Equations for adults aged 60–74 years were used to calculate the participants BMR values; 0.0386*body weight (kg) + 2.88, for women and 0.0499*body weight (kg) + 2.93, for men [31]. For individuals in energy balance, their EI/BMR value should equal their physical activity level (PAL). A PAL value of 1.35*BMR is considered the minimum energy expenditure compatible with a normal active lifestyle [34]. An EI/BMR value of 1.35 was therefore set as the cut-off value to determine potential underreporting of energy intake in this study [33, 34].

Percentages of energy (E%) from carbohydrate-, protein-, fat-, fibre- and alcohol intake were calculated. Recommended intervals of E% intake, from each macronutrient, were based on the NNR 2012 recommendations [8].

An alcohol intake of 10 g/day for women, and 20 g/day for men, was set as cut-off values to investigate the proportions that had an intake above recommended intake [8].

Protein intake/kg body weight and day was calculated by dividing gram of protein intake/day with body weight (BW) in kg [35]. A protein intake of 1.2 g/kg BW was set as cut-off value to investigate the proportions above recommended intake [8].

Calculations of energy from fibre has varied during the last five decades (from 0 to 4 kcal/gram fibre) [36]. In this study, energy from fibre was calculated according to the National Food Agency’s (NFA) database [37] by multiplying gram of fibre intake with 1.91 kcal, for all birth cohorts.

The NNR 2012 recommended intake values (RI) of vitamins and minerals include safety margins for individual variations. However, intakes between average requirement (AR) and RI levels are considered adequate [8]. AR levels for vitamin- and mineral intake were set as cut-off values to assess the risk for inadequate intake in this study.

Registered food intake was divided into 35 food groups (Additional file 1) in accordance with the NFA’s study on dietary patterns [38], with a special focus on health-related foods such as vegetables and pulses, fruits and berries, nuts and seeds, fish and shellfish, red and processed meat, dairy products, cereals, sweets, sweet beverages and alcohol intake [8].

Intake of nutritional supplements was not included in this study.

#### Sample characteristics

Educational level was dichotomized into compulsory primary education versus more than that (6 years in those born 1901–02, 1911–12 and 1922, 7 years in those born 1930, and 9 years in those born 1944). Smoking was dichotomized into smoker (current) or non-smoker (never smoked or past smoker). Living situation was dichotomized into institutionalized (hospitalized or otherwise institutionalized) or living in a home environment (i.e. own home or as a lodger). Marital status was dichotomized into married (currently married and/or cohabiting) or not married (never been married/not cohabiting, divorced, widowed). Physical activity level was divided into three groups; sedentary lifestyle (sedentary/low physical activity level), moderate physical activity level (low to moderate), high activity level (moderate to high). Body mass index (BMI) was calculated based on measured height and weight values (kg/m^2^).

The Strobe-Nut checklist was used during the writing process, see Additional file 2 [39, 40].

### Statistical analysis

All analyses were calculated stratified by sex. Normality tests were conducted using Shapiro-Wilk test, histogram and Normal Q-Q plot tests.

Values were expressed as means and standard deviations. General linear regressions were performed to examine time trends during the study period, with mean BMI, EI/BMR, E% intake from macronutrient-, energy-, nutrient- and food group values as dependent variables and years of each birth cohort as independent variables. Student’s t-test was performed to compare mean values of BMI, EI/BMR, E% intake from macronutrients, and energy-, nutrient-, and food intake levels between birth cohorts. The distribution of some of the variables was not normal, and for this reason alcohol-, micronutrient- and food intake values were log-transformed (Lg10) to normalise the data. For transforming alcohol and food intake, a value of 1 g was added to zero for foods never consumed. The log-transformed data was used in the linear regression analyses.

Birth cohort 1901–02 was excluded in the analyses of alcohol intake, since questions about beer, wine and spirits were not comparable in 1971–72. Beer intake was included in energy, alcohol and nutrient group means in 1971–72, but wine and spirit intake was not (no data of gram intake). Birth cohort 1944 was excluded in the analyses of margarine-, butter- and vegetable oil intake, since cooking fat was included in prepared dishes in 2014–16 and not registered separately as in previous cohorts. Cooking fat was included in energy and nutrient group means for all birth cohorts.

Chi-square tests were performed to evaluate potential differences in the following characteristics: the distribution of participants with an E% intake from macronutrients within the NNR 2012 recommendations, the distribution of participants with a micronutrient intake above AR; and the distribution of consumers in each food group.

To investigate effects of potential underreporting of energy intake, additional sensitivity analyses were performed by excluding participants with an EI/BMR < 1. 35 in the linear regressions of macro- and micronutrients.

Pearson correlation analyses were performed between EI/BMR and BMI values within each birth cohort.

The statistical analyses were performed using IBM SPSS STATISTICS 24. All statistical tests were two-tailed and *p*-values of < 0.05 were considered statistically significant.

## Results

### Characteristics of participants

Sample characteristics are given in Table 1. There was a linear increase in mean BMI (from 25 to 26) among men during the study period (*p* = 0.012), and an increase in BMI (from 26 to 27) between 1971 and 72 and 2000–02 among women (*p* = 0.019). However, BMI among both women (p = < 0.0001) and men (*p* = 0.038) decreased between 2000-02 and 2014–16 (from 27 to 26). Prevalence of smoking decreased (from 12 to 9% among women and 46 to 7% among men), physical activity level increased (those with a sedentary life style decreased from 20 to 3% among women and 12 to 3% among men) and educational level increased (those with only compulsory education or less decreased from 84 to 11% among women and 81 to 15% among men), during the study period. The majority of participants were community-dwelling (0 to 4% were institutionalized during the study period). The proportion married increased among women (from 47 to 60%), but not among men (79–81%), during the study period.

**Table 1:** Sample characteristics of participants, stratified by sex and birth cohorts

Birth cohort	1901–02 (*n* = 370)	1911–12 (*n* = 262)	1922 (*n* = 199)	1930 (*n* = 554)	1944 (*n* = 861)	
Examination years	1971–72	1981–83	1992–93	2000–02	2014–16	
Women total n	188	130	136	321	474	Linear trend
BMI* (kg/m^2^)						P value
Mean ± (SD)	26,1 (4,2)	27,1 (4,6)	26,1 (4,3)	27,1 (4,5)	25,6 (5,0)	0.084
	% (no. of cases/total cases)	% (no. of cases/total cases)	% (no. of cases/total cases)	% (no. of cases/total cases)	% (no. of cases/total cases)	χ^2^ P value
Living situation						
Institutionalized	4 (7/188)	0 (0/130)	2 (3/136)	1 (2/317)	1 (6/465)	0.029
Physical activity level						
Sedentary lifestyle	20 (38/187)	31 (39/128)	22 (30/136)	8 (25/314)	3 (14/467)	< 0.0001
Smoking						
Current smoker	12 (22/188)	12 (16/130)	24 (32/135)	15 (48/316)	9 (41/471)	0.0001
Educational level						
≤ Primary school	84 (157/186)	78 (101/129)	62 (80/129)	64 (202/317)	11 (50/469)	< 0.0001
Marital status						
Married	47 (88/188)	52 (68/130)	51 (69/136)	50 (158/317)	60 (280/467)	0.009
Men total n	182	132	63	233	387	Linear trend
BMI* (kg/m^2^)						P value
Mean ± (SD)	25,5 (3,3)	25,8 (3,6)	26,9 (3,9)	26,9 (3,9)	26,2(4,0)	0.012
	% (no. of cases/total cases)	% (no. of cases/total cases)	% (no. of cases/total cases)	% (no. of cases/total cases)	% (no. of cases/total cases)	χ^2^ P value
Living situation						
Institutionalized	2 (3/182)	0 (0/132)	3 (2/63)	0 (0/232)	2 (6/386)	0.102
Physical activity level						
Sedentary lifestyle	12 (21/182)	15 (20/130)	18 (11/63)	10 (22/226)	3 (10/381)	< 0.0001
Smoking						
Current smoker	46 (84/182)	33 (43/132)	25 (16/63)	14 (33/229)	7 (26/387)	< 0.0001
Education						
≤ Primary school	81 (147/181)	71 (94/130)	53 (18/34)	57 (132/231)	15 (59/382)	< 0.0001
Marital status						
Married	79 (142/181)	76 (100/132)	68 (43/63)	74 (173/232)	81 (310/382)	0.112

*There were missing body mass index (BMI) data for four men in 1992–93 and one in 2000–02, among women, there were missing BMI data for one individual in 1981–83, six in 1992–93, three in 2000–02 and two in 2014–16

### Time trends of energy- and nutrient intake and EI/BMR values

Mean energy-, macro- and micronutrient intake and EI/BMR values, in relation to birth cohorts, are given in Table 2. Mean EI/BMR values were above the cut-off value of 1.35 among both sexes at all examinations. There was a negative correlation between BMI and EI/BMR values (correlation range between − 0.324, r^2^ 0.11 and − 0.535, r^2^ 0.29, *p* = 0.01) among both sexes during the study period, indicating that participants with a higher BMI were more likely than other participants to underreport their energy intake.

**Table 2:** Time trends of EI/BMR-values, mean energy- and nutrient intake per day and comparisons between birth cohorts

Birth cohort	1901–02	1911–12	1922	1930	1944		
Examination years	1971–72	1981–83	1992–93	2000–02	2014–16		
Women	*n* = 188	*n* = 130	*n* = 136	*n* = 321	*n* = 474	Linear trend	Recommended daily intake*
Daily intake (except EI/BMR)	Mean (SD)	Mean (SD)	Mean (SD)	Mean (SD)	Mean (SD)	*P* value	
EI/BMR^1^	1.44 (0.44)	1.50 (0.40)	1.44 (0.38)	1.48 (0.38)	1.52 (0.37)ͣ ʲ ʳ	0.022	
Energy (kcal)	1869 (512)	1974 (509)	1885 (458)	1970 (472)ª	2006 (453)ª ʲ	0.001	
Protein (g)	67 (17)	70 (19)	76 (18)ª ʰ	77 (19)ª ʰ	84 (22)ª ʰ ʲ ʳ	< 0.0001	
Protein (g/kg BW)^1^	1.03 (0.32)	1.04 (0.32)	1.13 (0.32)ª ʰ	1.11 (0.32)ª ʰ	1.24 (0.38)ª ʰ ʲ ʳ	< 0.0001	1,20 g/kg BW
Fat (g)	75 (25)	82 (25)ª	74 (25)ʰ	76 (26)ʰ	83 (26)ª ʲ ʳ	0.001	
Carbohydrates (g)	219 (65)	226 (64)	213 (58)	226 (61)ʲ	202 (59)ª ʰ ʳ	0.0001	
Alcohol (g)^2^	1 (2)	2 (4)	4 (7)	5 (6)ʰ	9 (10) ʰ ʲ ʳ	< 0.0001	< 10
Fibre (g)	16 (5)	19 (6)ª	20 (7)ª	21 (7)ª ʰ ʲ	25 (8)ª ʰ ʲ ʳ	< 0.0001	25–30
Vitamin D (μg)	5.6 (2.2)	5.1 (1.8)ª	6.4 (3.0)ª ʰ	6.5 (2.8)ª ʰ	8.3 (3.8)ª ʰ ʲ ʳ	< 0.0001	10
Vitamin C (mg)	93 (55)	83 (44)	112 (70)ª ʰ	128 (68)ª ʰ ʲ	152 (78)ª ʰ ʲ ʳ	< 0.0001	75
B12 (μg)	10.0 (6.0)	7.6 (5.5)ª	9.5 (9.0)ʰ	6.5 (4.0)ª ʲ	6.5 (3.0)ª ʰ ʲ	< 0.0001	2
Folate (μg)	207 (63)	211 (61)	250 (100)ª ʰ	264 (79)ª ʰ	347 (114)ª ʰ ʲ ʳ	< 0.0001	300
Iron (mg)	13 (4)	12 (4)ª	12 (4)	12 (3)	12 (3)ª	0.077	9
Calcium (mg)	928 (348)	1025 (367)ª	1058 (374)ª	1055 (393)ª	1028 (399)ª	0.003	800
Men	*n* = 182	*n* = 132	*n* = 63	*n* = 233	*n* = 387		
EI/BMR^1^	1.42 (0.35)	1.59 (0.43)ª	1.45 (0.45)ʰ	1.56 (0.42)ª	1.40 (0.34)ʳ	0.095	
Energy (kcal)	2290 (546)	2555 (644)ª	2402 (707)	2603 (659)ª ʲ	2358 (519)ʰ ʳ	0.538	
Protein (g)	80 (20)	86 (22)ª	94 (26)ª ʰ	98 (25)ª ʰ	95 (23) ª ʰ	< 0.0001	
Protein (g/kg BW)^1^	1.06 (0.28)	1.13 (0.34)ª	1.16 (0.33)ª	1.21 (0.35)ª	1.16 (0.33)ª	0.0004	1,20 g/kg BW
Fat (g)	92 (27)	106 (34)ª	91 (30)ʰ	101 (36)ª	96 (29)ʰ	0.597	
Carbohydrates (g)	268 (70)	288 (76)ª	274 (101)	289 (83)ª	236 (67) ª ʰ ʲ ʳ	< 0.0001	
Alcohol (g)^2^	5 (5)	9 (13)	9 (11)	13 (15)ʰ ʲ	17 (17)ʰ ʲ ʳ	< 0.0001	< 20
Fibre (g)	19 (6)	22 (8)ª	22 (8)ª	24 (8)ª ʰ	27 (9) ª ʰ ʲ ʳ	< 0.0001	25–30
Vitamin D (μg)	6.7 (2.5)	6.4 (2.5)	8.1 (3.2)ª ʰ	8.4 (3.5)ª ʰ	9.6 (4.7) ª ʰ ʲ ʳ	< 0.0001	10
Vitamin C (mg)	85 (45)	84 (42)	100 (56)ʰ	129 (80)ª ʰ ʲ	147 (85) ª ʰ ʲ ʳ	< 0.0001	75
B12 (μg)	10.9 (6.1)	8.0 (4.3)ª	12.8 (9.2)ʰ	8.7 (5.8)ª ʲ	7.8 (4.1) ª ʲ ʳ	< 0.0001	2
Folate (μg)	230 (59)	245 (74)	268 (83)ª	301 (107)ª ʰ ʲ	356 (116) ª ʰ ʲ ʳ	< 0.0001	300
Iron (mg)	15 (5)	16 (5)	16 (6)	16 (4)	13 (3) ª ʰ ʲ ʳ	< 0.0001	9
Calcium (mg)	1051 (393)	1217 (450)ª	1170 (400)ª	1312 (557)ª ʲ	1059 (411)ʰ ʲ ʳ	0.901	800

*Based on the Nordic nutrition recommendations 2012

^1^There were missing protein/kgBW (body weight) and EI/BMR values, due to missing weight data, for one women in birth cohort 1911, five in birth cohort 1922, three in birth cohort 1930, two in birth cohort 1944 and four men in birth cohort 1922 and one man in birth cohort 1930

^2^Linear trends in alcohol intake are based on data from 1981 to 2016

ªSignificant difference compared with birth cohort1901–02

ʰSignificant difference compared with birth cohort 1911–12

ʲSignificant difference compared with birth cohort 1922

ʳSignificant difference compared with birth cohort1930

There was a linear increase in mean energy intake among women during the study period, while there was an increase between 1971 and 72 and 2000–01 (*p* < 0.0001) and a decrease between 2000 and 02 and 2014–16 among men (p < 0.0001).

Regarding macronutrients, there was a linear increase in mean protein intake, protein intake/kg BW and fibre intake, and a decrease in carbohydrate intake among both sexes, during the study period. The change in carbohydrate intake was mainly due to a decreased intake between 2000-02 and 2014–16 (p < 0.0001). There was a linear increase in mean fat intake among women, but not among men.

The proportion with a recommended intake of more than 1.2 g protein/kg BW was 28% in 1971–72, 26% in 1981–83, 31% in 1992–93, 32% in 2000–02, and 48% in 2014–16 among women. Among men, the proportions were 28% in 1971–72, 36% in 1981–83, 41% in 1992–93, 47% in 2000–02, and 37% in 2014–16.

Regarding micronutrients, there was a linear increase in mean folate-, vitamin D- and C intake, and a decrease in vitamin B12 intake among both sexes, during the study period. There was a linear decrease in mean iron intake among men, but not among women, and a linear increase in mean calcium intake among women, but not among men.

Results from sensitivity analyses, excluding participants with EI/BMR values < 1. 35, indicated the same linear trends for differences in macro- and micronutrient intake between birth cohorts as for the total population, except for fat-, calcium- (trends were no longer significant) and iron (decreased, p = < 0.0001) intake among women.

The distribution of participants that were more likely to have an adequate micronutrient intake above average requirement (>AR) is shown in Table 3. The proportion of participants with an >AR intake of vitamin D, vitamin C and folate increased among both sexes during the study period. The proportion participants with an >AR intake of calcium increased among women, but not among men. The majority of participants (range 96–100%) had a B12 and iron intake >AR at all examinations, among both sexes.

**Table 3:** Distribution of participants with a micronutrient intake above average requirement intake (>AR)

Birth cohort		1901–02	1911–12	1922	1930	1944			1901–02	1911–12	1922	1930	1944	
Examination years		1971–72	1981–83	1992–93	2000–02	2014–16			1971–72	1981–83	1992–93	2000–02	2014–16	
	Women	n = 188	n = 130	n = 136	n = 321	n = 474	χ^2^	Men	n = 182	n = 132	n = 63	n = 233	n = 387	χ^2^
	>AR	n (%)	n (%)	n (%)	n (%)	n (%)	P value	>AR	n (%)	n (%)	n (%)	n (%)	n (%)	*P* value
Vitamin D (μg)	> 7.5 μg	36 (19)	16 (12)	32 (23)	94 (29)	244 (51)	< 0.0001	> 7.5 μg	61 (33)	38 (29)	29 (46)	127 (54)	262 (68)	< 0.0001
Vitamin C (mg)	> 50 mg	147 (78)	100 (77)	113 (83)	289 (90)	452 (95)	< 0.0001	> 60 mg	119 (65)	81 (61)	45 (71)	192 (82)	348 (90)	< 0.0001
B12 (μg)	> 1.4 μg	188 (100)	129 (99)	135 (99)	320 (99)	474 (100)	0.334	> 1.4 μg	182 (100)	132 (100)	63 (100)	233 (100)	387 (100)	0.839
Folate (μg)	> 200 μg	93 (49)	69 (53)	87 (64)	253 (79)	448 (94)	< 0.0001	> 200 μg	127 (70)	93 (70)	51 (81)	205 (88)	365 (94)	< 0.0001
Iron (mg)	> 6 mg	184 (98)	126 (97)	135 (99)	318 (99)	457 (96)	0.094	> 7 mg	178 (98)	128 (97)	63 (100)	232 (99)	380 (98)	0.260
Calcium (mg)	> 500 mg	170 (90)	124 (95)	132 (97)	312 (97)	463 (98)	0.0003	> 500 mg	171 (94)	127 (96)	60 (95)	228 (98)	377 (97)	0.187

Mean E% intake and E% distribution of macronutrients and alcohol are given in Table 4. There was a linear increase in mean E% intake from protein, alcohol and fibre, and a decrease in E% intake from carbohydrates among both sexes, during the study period. There was no linear trend for changed mean E% intake from fat, either among women or men. The proportions with an E% intake according to the NNR 2012 recommendations, increased for protein intake, and decreased for fat-, carbohydrate- and alcohol intake among both sexes, during the study period.

**Table 4:** Distribution of energy percent (E%) intake from protein, fat, carbohydrate, fibre and alcohol

Birth cohort	1901–02		1911–12		1922		1930		1944				
Examination years	1971–72		1981–83		1992–93		2000–02		2014–16				
Women	n = 188	*within NNR rec.*	n = 130	*within NNR rec.*	n = 136	*within NNR rec.*	n = 321	*within NNR rec.*	n = 474	*within NNR rec.*	Linear trend	*χ*^*2*^ *within NNR rec.*	Recommended E% intake*
	Mean (SD)	*%*	Mean (SD)	*%*	Mean (SD)	*%*	Mean (SD)	*%*	Mean (SD)	*%*	P value	*P value %*	
Protein E%	15 (2)	*33*	14 (2)	*33*	16 (3)ª ʰ	*57*	16 (3)ª ʰ	*52*	17 (3)ª ʰ ʲ ʳ	*60*	< 0.0001	*< 0.0001*	15–20
Fat E%	36 (6)	*76*	37 (5)	*72*	35 (6)ʰ	*77*	34 (6)ª ʰ	*77*	37 (7)ª ʲ ʳ	*67*	0.132	*0.007*	25–40
Carbohydrates E%	47 (6)	*65*	46 (5)ª	*55*	45 (6)ª	*53*	46 (6)ª	*58*	40 (7)ª ʰ ʳ ʲ	*23*	< 0.0001	*< 0.0001*	45–60
Alcohol E%	0 (1)	*100*	1 (1)ª	*96*	2 (3)ª	*96*	2 (2)ª ʰ	*92*	3 (4)ª ʰ ʲ ʳ	*79*	< 0.0001	*< 0.0001*	< 5
Fibre E%	2 (1)		2 (0.5)		2 (1)		2 (1)		3 (1)		< 0.0001		No rec.
Men	n = 182		n = 132		n = 63		n = 233		n = 387				
Protein E%	14 (2)	*30*	14 (2)ª	*20*	16 (3)ª ʰ	*49*	15 (2)ª ʰ	*54*	16 (3)ª ʰ ʳ	*61*	< 0.0001	*< 0.0001*	15–20
Fat E%	36 (5)	*84*	37 (5)ª	*70*	34 (6)ʰ	*81*	34 (6)ª ʰ	*78*	37 (6)ʲ ʳ	*73*	0.939	*0.021*	25–40
Carbohydrates E%	47 (5)	*64*	45 (5)ª	*49*	45 (7)ª	*44*	45 (7)ª	*49*	40 (7)ª ʰ ʲ ʳ	*25*	< 0.0001	*< 0.0001*	45–60
Alcohol E%	1 (2)	*95*	2 (3)ª	*86*	3 (3)ª	*84*	4 (4)ª ʰ ʲ	*76*	5 (5)ª ʰ ʲ	*61*	< 0.0001	*< 0.0001*	< 5
Fibre E%	2 (1)		2 (1)		2 (0.5)		2 (1)		2 (1)		< 0.0001		No rec.

* Based on the Nordic nutrition recommendations (NNR) 2012

ªSignificant difference compared with birth cohort1901–02

ʰSignificant difference compared with birth cohort 1911–12

ʲSignificant difference compared with birth cohort 1922

ʳSignificant difference compared with birth cohort1930

ʸNNR recommended intake (RI) levels of macro- and micronutrients

### Time trends in food- and beverage intake

Mean food and beverage intake, in relation to birth cohorts, are given in Table 5. There was a linear increase in intake of vegetables and pulses, fruits and berries, nuts and seeds, fish and shellfish among both sexes, during the study period. There was a linear increase in red and processed meat intake among women during the study period, while there was an increase only between 1971 and 72 and 2000–02 among men (*p* < 0.0001). However, mean intake of red and processed meat decreased between 2000-02 and 2014–16, among both women (*p* = 0.004) and men (*p* = 0.012). There was a linear decrease in intake of keyhole (low-fat/low sugar) milk products (milk, sour milk and yoghurt) and non-keyhole (semi/full-fat and sweetened) milk products among women, during the study period. This trend was not observed among men. However, the intake of keyhole milk products decreased between 2000-02 and 2014–16 among men (p < 0.0001). There was a linear increase in intake of cream and crème fraiche, cheese, pasta, rice and food grains and fibre-rich (> 5% fibre) bread, and a decrease in intake of cereals and refined bread (≤5% fibre) among both sexes, during the study period.

**Table 5:** Time trends and comparisons between birth cohorts in food and beverage intake

Birth cohort	1901–02	1911–12	1922	1930	1944	
Examination years	1971–72	1981–83	1992–93	2000–02	2014–16	
Women	n = 188	n = 130	n = 136	n = 321	n = 474	Linear trend
	Mean (SD) gram/day	Mean (SD) gram/day	Mean (SD) gram/day	Mean (SD) gram/day	Mean (SD) gram/day	P value
Fish and shellfish/seafood	46 (22)	31 (20)ª	54 (35)ª ʰ	54 (36)ª ʰ	65 (43)ª ʰ ʲ ʳ	< 0.0001
Meat and processed meat	66 (39)	78 (42)ª	84 (45)ª	81 (41)ª	69 (40)ʰ ʲ ʳ	0.029
Poultry	8 (8)	7 (6)	8 (9)	12 (13)ª ʰ ʲ	22 (25)ª ʰ ʲ ʳ	< 0.0001
Eggs	26 (21)	20 (12)ª	20 (19)ª	17 (16)ª	26 (21)ʰ ʲ ʳ	0.074
Potatoes	129 (69)	124 (65)	95 (58)ª ʰ	97 (51)ª ʰ	81 (66)ª ʰ ʲ ʳ	< 0.0001
Vegetables and pulses	53 (41)	56 (42)	143 (113)ª ʰ	145 (94)ª ʰ	205 (123)ª ʰ ʲ ʳ	< 0.0001
Fruits and berries	155 (116)	129 (83)ª	182 (126)ʰ	205 (134)ª ʰ	260 (157)ª ʰ ʲ ʳ	< 0.0001
Keyhole milk products	147 (232)	162 (207)	146 (221)	165 (211)	78 (201)ª ʰ ʲ ʳ	0.007
Non-Keyhole milk products	204 (225)	248 (237)	205 (232)	184 (222)ʰ	189 (182)ʰ	0.001
Cream and crème fraîche	17 (21)	23 (23)ª	18 (20)ʰ	23 (23)ª ʲ	36 (45)ª ʰ ʲ ʳ	< 0.0001
Cheese	28 (19)	32 (21)	43 (36)ª ʰ	46 (37)ª ʰ	49 (45)ª ʰ	< 0.0001
Fast food	0 (0)	0 (2)	0 (3)ª	2 (6)ª ʰ ʲ	3 (9)ª ʰ ʲ ʳ	< 0.0001
Pasta, rice and food grain	7 (10)	17 (34)ª	22 (22)ª	31 (31)ª ʰ ʲ	35 ((43)ª ʰ ʲ	< 0.0001
Bread refined	61 (50)	48 (52)ª	37 (46)ª	35 (41)ª ʰ	24 (34)ª ʰ ʲ ʳ	< 0.0001
Bread fibre-rich	36 (40)	65 (51)ª	54 (38)ª	65 (45)ª ʲ	58 (41)ª ʳ	< 0.0001
Cereals	92 (98)	94 (87)	63 (74)ª ʰ	40 (52)ª ʰ ʲ	42 (58)ª ʰ ʲ	< 0.0001
Savoury bakery	11 (13)	9 (9)	11 (14)	12 (16)ʰ	12 (14)ʰ	0.005
Sweet bakery	54 (44)	53 (45)	35 (29)ª ʰ	34 (31)ª ʰ	21 (24)ª ʰ ʲ ʳ	< 0.0001
Desserts	62 (65)	73 (88)	48 (68)ʰ	53 (62)ʰ	49 (71)ª ʰ	0.003
Sweet condiments	34 (25)	34 (31)	27 (28)ª	21 (22)ª ʰ ʲ	14 (18)ª ʰ ʲ ʳ	< 0.0001
Sweets, candy and chocolate	6 (10)	5 (7)	9 (19)ʰ	11 (12)ª ʰ	12 (18)ª ʰ	< 0.0001
Salads	0 (0)	0 (0)	1 (7)	0 (2)ª ʰ	4 (19)ª ʰ ʲ ʳ	< 0.0001
Soups	33 (22)	42 (28)ª	40 (48)	50 (53)ª	29 (40)ʰ ʲ ʳ	< 0.0001
Sauces and condiments	0 (0)	0 (0)	5 (12)ª ʰ	9 (15)ª ʰ ʲ	25 (29)ª ʰ ʲ ʳ	< 0.0001
Substitute products	0 (0)	0 (0)	1 (5)ª ʰ	0 (5)	17 (77)ª ʰ ʲ ʳ	< 0.0001
Margarine^	17 (13)	25 (15)ª	22 (21)ª	21 (17)ª ʰ	12 (12)	0.045
Butter^	8 (12)	5 (10)ª	3 (8)ª	3 (9)ª ʰ	1 (4)	< 0.0001
Vegetable oil^	0 (1)	0 (0)ª	1 (2)ª ʰ	3 (5)ª ʰ ʲ	2 (6)	< 0.0001
Snacks	0 (0)	0 (0)	0 (1)ª ʰ	1 (2)ª ʰ ʲ	2 (8)ª ʰ ʲ ʳ	< 0.0001
Nuts and seeds	0 (0)	0 (0)	2 (6)ª ʰ	1 (4)ª ʰ	12 (17)ª ʰ ʲ ʳ	< 0.0001
Juice	29 (62)	37 (72)	57 (119)ª	68 (99)ª ʰ	49 (85)ª ʳ	< 0.0001
Coffee	437 (177)	508 (246)ª	499 (280)ª	473 (320)	423 (338)ʰ ʲ ʳ	< 0.0001
Tea	146 (59)	169 (82)ª	122 (312)	136 (200)ʰ	195 (280)ª ʲ ʳ	< 0.0001
Soda	122 (208)	68 (110)ª	57 (124)ª	79 (179)ª	33 (92)ª ʰ ʲ ʳ	< 0.0001
Alcoholic beverages*	46 (75)	49 (81)	63 (99)	90 (109)ʰ ʲ	107 (127)ʰ ʲ ʳ	< 0.0001
Men	n = 182	n = 132	n = 63	n = 233	n = 387	
Fish and shellfish/seafood	58 (35)	43 (25)ª	69 (39)ª ʰ	65 (44)ª ʰ	77 (57)ª ʰ ʳ	< 0.0001
Meat and processed meat	80 (30)	96 (47)ª	125 (65)ª ʰ	110 (49)ª ʰ ʲ	97 (51)ª ʲ ʳ	0.119
Poultry	7 (7)	6 (7)	5 (7)	13 (14)ª ʰ ʲ	22 (21)ª ʰ ʲ ʳ	< 0.0001
Eggs	31 (21)	32 (33)	25 (20)	23 (24)ª ʰ	30 (24)ʳ	0.462
Potatoes	202 (105)	209 (95)	156 (91)ª ʰ	136 (65)ª ʰ	125 (91)ª ʰ ʲ	< 0.0001
Vegetables and pulses	44 (34)	47 (47)	96 (63)ª ʰ	129 (101)ª ʰ ʲ	186 (141)ª ʰ ʲ ʳ	< 0.0001
Fruits and berries	132 (95)	130 (100)	142 (89)	178 (138)ª ʰ ʲ	228 (173)ª ʰ ʲ ʳ	< 0.0001
Keyhole milk products	118 (235)	171(283)	158 (255)	162 (253)	59 (144) ª ʰ ʲ ʳ	0.194
Non-Keyhole milk products	267 (273)	313 (278)	219 (239)ʰ	259 (320)	209 (235)ª ʰ ʳ	0.253
Cream and crème fraîche	15 (21)	19 (22)	15 (21)	21 (20)ª	29 (31)ª ʰ ʲ ʳ	< 0.0001
Cheese	33 (23)	38 (30)	46 (42)ª	58 (46)ª ʰ	43 (31)ª ʳ	< 0.0001
Fast food	0 (0)	0 (3)	1 (4)	2 (7) ª ʰ	6 (13)ª ʰ ʲ ʳ	< 0.0001
Pasta, rice and food grain	8 (12)	14 (17)ª	23 (26)ª ʰ	35 (37)ª ʰ ʲ	45 (46)ª ʰ ʲ ʳ	< 0.0001
Bread refined	86 (64)	92 (68)	55 (56)ª ʰ	55 (51)ª ʰ	34 (44)ª ʰ ʲ ʳ	< 0.0001
Bread fibre-rich	43 (54)	57 (65)ª	76 (54)ª	78 (56)ª ʰ	75 (53)ª ʰ	< 0.0001
Cereals	106 (102)	102 (114)	98 (112)	58 (68)ª ʰ ʲ	48 (66)ª ʰ ʲ	< 0.0001
Savoury bakery	17 (15)	15 (14)	11 (13)ª ʰ	14 (19)	15 (21)	0.01
Sweet bakery	64 (60)	56 (46)	43 (41)ª	45 (35)ª ʰ	22 (25)ª ʰ ʲ ʳ	< 0.0001
Desserts	56 (67)	73 (76)ª	45 (54)ʰ	57 (73)ʰ	53 (71)ʰ	0.798
Sweet condiments	42 (26)	45 (34)	49 (58)	36 (38)ʰ	17 (20)ª ʰ ʲ ʳ	< 0.0001
Sweets, candy and chocolate	6 (9)	6 (9)	10 (15)	11 (14)ª ʰ	12 (18)ª ʰ	< 0.0001
Salads	0 (0)	0 (0)	0 (1)	1 (7)	4 (27)ª ʰ ʲ ʳ	< 0.0001
Soups	41 (34)	54 (43)ª	66 (139)	51 (60)ª	35 (53)ʰ ʳ	< 0.0001
Sauces and condiments	0 (0)	0 (0)	4 (14)ª ʰ	14 (19)ª ʰ ʲ	37 (36)ª ʰ ʲ ʳ	< 0.0001
Substitute products	0 (0)	0 (0)	0 (0)	0 (3)ª	9 (49)ª ʰ ʲ ʳ	< 0.0001
Margarine^	21 (17)	30 (21)ª	33 (20)ª	29 (25)ª	16 (15)	0.007
Butter^	11 (15)	11 (20)	2 (7)ª ʰ	3 (10)ª ʰ	2 (7)	< 0.0001
Vegetable oil^	1 (1)	0 (0)	1 (2)ª ʰ	3 (6)ª ʰ ʲ	2 (9)	< 0.0001
Snacks	0 (0)	0 (0)	0 (0)	1 (3)ª ʰ ʲ	2 (6)ª ʰ ʲ ʳ	< 0.0001
Nuts and seeds	0 (0)	0 (1)	0 (1)ª	2 (10)ª ʰ ʲ	10 (16)ª ʰ ʲ ʳ	< 0.0001
Juice	12 (37)	24 (62)	37 (88)ª	85 (134)ª ʰ ʲ	72 (112)ª ʰ ʲ	< 0.0001
Coffee	486 (229)	572 (254)ª	520 (363)	499 (348)ʰ	455 (358)ʰ	< 0.0001
Tea	162 (76)	191 (85)ª	129 (194)ʰ	170 (236)	163 (235)ʲ	< 0.0001
Soda	120 (199)	99 (129)	134 (222)	172 (318)ª ʰ	57 (138)ª ʰ ʲ ʳ	0.041
Alcoholic beverages*	183 (193)	164 (207)	160 (168)	231 (228)ʰ ʲ	237 (255)ʰ ʲ	< 0.0001

* Linear trends in intake of alcoholic beverages are based on data from 1981 to 2016

^ Linear trends in intake of margarine, butter and vegetable oil are based on data from 1971 to 2002

¶ Keyhole is the National Food Agency-labelling scheme, which guides healthy food choices. For milk and yogurt to meet the criteria for the Keyhole, fat content has to be limited to a maximum of 0.7%, and for flavoured products there is an additional limit for sugars: a maximum of 9%

ªSignificant difference compared with birth cohort1901–02

ʰSignificant difference compared with birth cohort 1911–12

ʲSignificant difference compared with birth cohort 1922

ʳSignificant difference compared with birth cohort1930

There was a linear increase in intake of sweets (e. g. candy, chocolate) and snacks (e. g. crisps, popcorn, cheese doodles), and a decrease in intake of sweet bakery (e. g. buns, cookies, cakes) and sweet condiments (e. g. sugar, honey, jam) among both sexes, during the study period. Among women, but not among men, there was also a decrease in intake of desserts (e. g. sweet pie, ice cream, chocolate mousse), during the study period.

There was a linear increase in juice (fruit- and vegetable juices) intake, and a decrease in soda (soft drinks, lemonade) intake among both sexes, during the study period. However, among women, there was a decrease in juice intake between 2000-02 and 2014–16, and the linear decrease in soda was mainly due to a decreased intake between 2000-02 and 2014–16, among both sexes (*p* < 0.0001).

The proportions of participants who consumed different foods and beverages are given in Table 6. Consumers of vegetables and pulses, fruits and berries, fish and shellfish, red and processed meat ranged from 94 to 100% during the study period, while consumers of nuts and seeds increased from 0 to 76% among women, and from 0 to 72% among men, during the study period. Consumers of keyhole milk products increased between 1971 and 72 and 2000–02 but decreased between 2000-02 and 2014–16 among both sexes. However, consumers of non-keyhole milk products, cream, crème fraiche and cheese increased among both sexes during the study period, especially consumers of cream and crème fraiche. Consumers of pasta, rice, food grains and fibre-rich bread increased during the study period, and consumers of refined bread decreased. Consumers of desserts, sweets and candy, snacks and juice increased, among both sexes during the study period, and consumers of soda increased among men, but not among women.

**Table 6:** Proportion consumers (%) of each food group and changes in proportions during the study period

	Women						Men					
Birth cohort	1901–02	1911–12	1922	1930	1944		1901–02	1911–12	1922	1930	1944	
Examination years	1971–72	1981–83	1992–93	2000–02	2014–16		1971–72	1981–83	1992–93	2000–02	2014–16	
	n = 188	n = 130	n = 136	n = 321	n = 474	χ^2^	n = 182	n = 132	n = 63	n = 233	n = 387	χ^2^
Food groups	%	%	%	%	%	P value	%	%	%	%	%	P value
Fish and shellfish/seafood	98	99	98	98	99	0.472	98	99	98	98	100	0.063
Meat and processed meat	100	100	97	98	98	0.186	100	98	100	99	99	0.243
Poultry	67	79	66	75	94	< 0.0001	67	67	52	74	92	< 0.0001
Eggs	81	89	86	89	94	< 0.0001	90	94	100	91	96	0.006
Potatoes	100	99	100	99	95	< 0.0001	100	99	100	98	98	0.555
Vegetables and pulses	98	99	98	100	100	0.021	94	98	98	100	100	< 0.0001
Fruits and berries	96	98	99	97	100	0.004	95	99	98	98	99	0.010
Keyhole milk products¶	40	45	47	61	34	< 0.0001	27	39	40	48	28	< 0.0001
Non-Keyhole milk products¶	60	71	75	75	82	< 0.0001	65	74	67	72	83	< 0.0001
Cream and crème fraîche	57	75	83	89	90	< 0.0001	52	65	68	82	84	< 0.0001
Cheese	87	95	96	96	98	< 0.0001	87	90	95	97	99	< 0.0001
Fast food	0	1	3	10	29	< 0.0001	0	1	3	12	33	< 0.0001
Pasta, rice and food grain	56	73	89	94	92	< 0.0001	54	64	83	88	94	< 0.0001
Bread refined	80	68	63	73	62	< 0.0001	81	84	73	83	66	< 0.0001
Bread fibre-rich	65	86	90	96	96	< 0.0001	67	79	87	94	94	< 0.0001
Cereals	80	85	85	86	79	0.049	79	80	78	84	82	0.659
Savoury bakery	62	75	68	69	83	< 0.0001	76	77	70	66	77	0.015
Sweet bakery	95	95	93	94	91	0.228	93	96	91	92	87	0.018
Desserts	80	99	88	89	90	< 0.0001	76	95	84	88	93	< 0.0001
Sweet condiments	94	93	93	94	93	0.912	94	96	92	94	95	0.813
Sweets, candy and chocolate	64	54	79	85	90	< 0.0001	61	59	68	71	82	< 0.0001
Salads	0	0	2	3	12	< 0.0001	0	0	2	2	8	< 0.0001
Soups	92	93	83	83	75	< 0.0001	88	92	89	83	76	< 0.0001
Sauces and condiments	0	0	43	70	91	< 0.0001	0	0	30	79	94	< 0.0001
Substitute products	0	0	5	2	17	< 0.0001	0	0	0	2	11	< 0.0001
Margarine^	87	95	93	95	78	0.014	90	91	100	94	76	0.035
Butter^	40	32	27	34	12	0.109	46	39	22	32	16	0.002
Vegetable oil^	4	0	21	58	23	< 0.0001	2	1	14	52	21	< 0.0001
Snacks	0	0	6	17	41	< 0.0001	0	0	0	13	36	< 0.0001
Nuts and seeds	0	0	15	22	76	< 0.0001	0	1	13	20	72	< 0.0001
Juice	22	35	43	60	46	< 0.0001	10	25	27	59	56	< 0.0001
Coffee	100	99	93	96	95	0.022	98	99	92	94	94	0.025
Tea	100	99	34	63	72	< 0.0001	98	99	38	60	71	< 0.0001
Soda	41	45	37	45	37	0.139	39	54	59	64	43	< 0.0001
Alcoholic beverages*	36	55	75	84	88	< 0.0001	75	83	86	92	92	< 0.0001

*Comparisons of consumers of alcoholic beverages are based on data from 1981 to 2016

^ Comparisons of consumers of margarine, butter and vegetable oils are based on data from 1971 to 2002

¶ Keyhole is the National Food Agency-labelling scheme, which guides healthy food choices. For milk and yogurt to meet the criteria for the Keyhole, fat content has to be limited to a maximum of 0.7%, and for flavoured products there is an additional limit for sugars: a maximum of 9%

### Alcohol intake

**Fig. 2:**
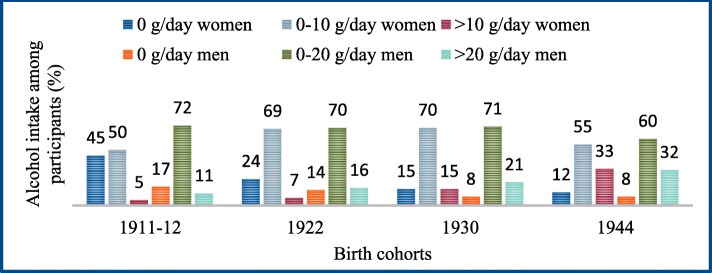
Distribution of alcohol intake (gram/day) among participants

There was a linear increase in mean gram intake of alcohol (Table 2) and alcoholic beverages (Table 5) among both sexes, during the study period (1981–2016). There was a linear increase in beer (> 2.8% alcohol) (*p* < 0.0001) and wine (p < 0.0001) intake, a decrease in low alcohol beer (< 2.8% alcohol) (*p* < 0.0001) intake, but no linear change in mean liquor intake, among both sexes. Consumers of alcoholic beverages increased among both sexes, during the study period (Table 6). The proportion with an average alcohol intake above 10 g/day for women and 20 g/day for men, was 5% in women and 11% in men in 1981–83, 7% in women and 16% in men in 1992–93, 15% in women and 21% in men in 2000–02, and 33% in women and 32% in men in 2014–16 (Fig. 2).

## Discussion

Dietary patterns and nutrient intake changed considerably among 70-year-olds between 1971 and 2016, with overall healthier dietary patterns, and a higher nutrient density in later born birth cohorts. Especially, there was an increase in healthful foods, such as fruits (including berries), vegetables and pulses, fish and shellfish, nuts and seeds. In addition, there was an increase in protein intake and a decrease in the proportion of participants at risk of an inadequate vitamin and mineral intake in later born birth cohorts. However, the intake of alcohol increased successively across examinations since 1981, especially among women.

Some changes in food intake were expected since urbanisation, globalisation, technical development and lifestyle changes have influenced food consumption in Sweden over the last half-century [41]. A relative decrease in food prices, a broadened supply of food products, a higher import of different foods, an increased access to foods, as well as changes in socio-economic factors such as a higher educational level and better economy, could be some explanations for changes in dietary intake during the study period [41–43]. The degenerative processes of ageing and disease can affect dietary intake negatively, with a decrease in food intake and changes in food choices as a consequence [44]. During the study period there has been an increase in average life expectancy from 77 to 84 years in women and from 72 to 81 years in men [45]. This increase could indicate that the birth cohort of 70 year olds examined in 2014–16 might have been healthier and less affected by the negative consequences of the ageing process and disease, and therefore more able to maintain a healthier dietary intake than 70-year olds examined in 1971. The increased media access and use of internet between 2000-02 and 2014–16 might have affected trends in dietary intake. In addition, there has been an increase in dietary cooking magazines and television shows during this period, as well as an increase in dietary articles in traditional newspapers. These sources give dietary advice, which are both in line with, and opposed to, official guidelines. Thus, many of the changes are probably due to period effects, rather than an effect of birth cohort and life-long dietary habits. Functional ability, including the ability to buy and prepare food, could also have an impact on dietary intake, and a previous H70 study, comparing 75-year olds born 30 years apart (1976–2006), showed that later born cohorts were less dependent in activities of daily living (ADL) and more engaged in leisure activities compared with earlier cohorts [46].

The increased intake of vegetables, fruit, meat, fish, cream, cheese, beer, wine, candy and snacks, and the decreased intake of milk, cereals, potatoes, sweet bakery and sugar was in line with the Swedish Board of Agriculture’s report on food consumption in the Swedish population over the last 50 years [41], and with the National Food Agency’s (NFA) population-based study “Riksmaten 2010–11” in Sweden [36]. However, the decrease in cereal intake might have been affected by changes in type of cereals consumed, from those containing more water, such as gruel, to dry foods such as muesli and bran flakes that weigh less.

The intake of certain healthful foods, such as fruits and vegetables, fish and seafood, nuts and seeds was highest in 2014–16, and consequently the proportion individuals at risk of insufficient vitamin-and mineral intakes, was lowest in those years. A study of dietary intake among US adults, examined 1999–2012, showed similar trends among older adults (> 65 years), with increased intakes of whole grain products, nuts and seeds, fish and shellfish, cheese and protein and decreased intakes of milk, sugars and carbohydrates [47]. Dietary trends during the latter part of the study period, especially 2014–16, increasingly correspond to Mediterranean food patterns in the EPIC (EPIC-Elderly cohort) and SENECA studies [48, 49], where the southern European countries had a higher intake of fruits, vegetables, legumes, fish, wine and vegetable oils and a lower consumption of dairy products, sugar and cakes, than the northern European countries. Other studies have shown that high quality diets are related to higher education, non-smoking and greater physical activity [36, 48, 49]. This might have influenced our findings as greater proportions of participants in 2014–16 were non-smoking, highly educated and physically active, compared to earlier-born cohorts.

Mean energy intake increased during the study period, despite a shift towards a decreased energy intake among men between 2000-02 and 2014–16. The decrease in energy intake among men in 2014–16 might partially be due to underreporting of energy intake, since EI/BMR values between 2000-02 and 2014–16 also decreased. However, among women, the mean EI/BMR value was highest in 2014–16. Some misreporting of energy intake can be expected when examining dietary intake in a population, and underreporting of energy intake is common [31, 50–52]. Results from our study indicated that individuals with a higher BMI were more likely to underreport their energy intake, a result that is in line with other studies in older adults [53, 54]. However, mean EI/BMR values were above the cut-off value (1.35) for potential underreporting of energy intake during the study period, and the sensitivity analyses, including only individuals with a EI/BMR value of > 1.35, supported the overall time trends of differences in nutrient intake.

Mean energy intake, and E% intake from macronutrients, were in line with the EPIC study, which examined older adults from nine European countries between 1992 and 2000 [48]. The Swedish longitudinal Västerbotten intervention programme (VIP) study, which examined dietary intake in a population from northern Sweden between 1996 and 2014, also exhibited similar patterns in E% changes from macronutrient intake over a 10-year period (in four different age groups; 30, 40, 50 or 60 years at baseline) [55]. During the time period between 2000-02 and 2014–16, the low-carbohydrate and high fat diet was popular in Sweden [41, 56]. This trend could potentially explain the decrease in E% intake from carbohydrates and the increase in E% intake from fat between 2000-02 and 2014–16, which negatively affected compliance to the E% intake recommendations for carbohydrates and fat in 2014–16 [8]. The decrease in E% intake from carbohydrates between 2000-02 and 2014–16 indicates changes in preferences towards fewer carbohydrate dense foods, e.g. less refined bread, cakes, buns and soda and more vegetables, pulses, fruits and berries. The increased E% intake from fibre also indicates an increase in fibre-rich sources of carbohydrate intake, such as whole grain products and greens. The shift from key-hole milk products to non-keyhole milk products, together with the increase in cream and crème fraiche between 2000-02 and 2014–16, could also reflect this dietary trend [56].

The increase in alcohol intake was mainly due to an increase in wine and beer consumption, among both sexes. These results are in line with a previously published study examining risk consumption of alcohol, as reported from an interview by healthcare professionals, in the same population [57]. The results are also in line with “Riksmaten 2010–11” [36] and a report of self-reported alcohol intake in Sweden and might suggest a more continental and “quality of life” way of drinking [58, 59] in later born birth cohorts, which also partly follows other changes in dietary intake. A British study showed that alcohol intake among older adults decreased with deteriorating health, but increased among older adults with higher income and education [60]. The negative side effects of a higher alcohol intake could be an increased risk for disease, falls and fractures [61–63]. The National Board of Health and Welfare in Sweden recently reported that deaths associated with alcohol increased during the twenty-first century among older adults in Sweden, especially among women [64].

A higher protein intake could be beneficial in preventing and/or delaying age-related sarcopenia [65, 66]. The PROMISS-study has examined protein intake in community-dwelling older adults, and results showed that a low protein (≤ 1.0 g/kg BW/day) intake is common among older adults [67, 68]. In our study, mean intake of protein and protein-rich foods such as fish, poultry and meat increased during the study period, but despite this there was still more than 50% who had a protein intake below the recommended level of 1.2 g/kg BW/day. These results indicate potential benefits of promoting healthy protein intake during the ageing process.

The higher nutrient density of vitamins and minerals in the later born birth cohorts were in line with Riksmaten 2010–11 [36]. The increase in participants having an intake of vitamin C and folate above requirements (AR) was most likely due to the increased fruit-, berries- and vegetable intake. However, there was a substantial proportion who did not reach the average requirement of 7.5 μg of vitamin D/day. Even less reached the recommended intake level of 10 μg/day, indicating that vitamin D supplements could be advisable in this age group (especially for those with a low intake of fish and fortified dairy products). However, the database used for the 2014–16 examination included values for vitamin D in fortified foods (e.g. dairy products) before values were increased in the late 2015 (our database was from January 2015) update of the national database [69]. Vitamin D intake from fortified foods might therefore be somewhat underestimated in the 2014–16 examination. In Sweden, flour was fortified with iron between 1944 and 1995. However, this has been taking into account when calculating iron intake in the 1971 to 1992–93 examinations.

A unique feature of this study is the fact that the dietary examination methodology has been comparable in all five birth cohorts, providing an opportunity to monitor dietary intake among older adults for a period of almost half a century. Other strengths are that the dietary examinations were performed by registered dietitians, and that the interviews were face-to face interviews, giving the participants an opportunity to describe their dietary intake in detail, in a way that would not be possible in a self-administered Food frequency questionnaire, (the most commonly used method in today’s large-scale epidemiologic studies) [40]. Further, the interviews started with a 24 h recall of a regular day, which gave the interviewer an opportunity to cross check that intake of foods eaten regularly were not missed in the interview.

The study is not without limitations. For instance, dietary data from 1971-72, 1981–83, 1992–93, and 2000–02 were calculated in the Swedish NFA’s nutrient database (PC-kost) after the 2000–02 examination [16]. When these calculations were done, there were several foods from the previous examinations (1971 to 1992–93) that were no longer listed in the 2000–02 database, so these were retrieved from earlier databases (based on the Swedish NFA’s nutrient database of the time). The 2014–16 dietary examination was calculated in an updated version of the Swedish NFA’s nutrient database 2015 [69]. However, we believe that the overall comparability between birth cohorts should not be affected, and that the benefits of using an updated database, including new foods and updates in nutrient content of foods, outweigh the disadvantages.

There is presently no dietary examination method that can measure exact intake in a larger population. There are individual differences in memory and recall, and in the ability to describe portion sizes and frequency. The latter is especially important in older populations. This uncertainty exists in most dietary examination methods [40], including ours. To capture new foods, the diet history method used in our study has been slightly modified between examinations. Questions about cooking fat were asked and registered somewhat differently in the 1944 birth cohort, as were alcohol intake in the birth cohort 1901–02, affecting comparability between birth cohorts. However, this should not affect overall comparability in dietary intake between cohorts to a significant degree. Further, participation rates varied between examinations, and the dietary examination was only performed in a smaller subsample in the first three examinations [16, 18], which might have affected the representability of the population.

With population ageing and growing, health promoting activities within public health are essential. This study provides an insight into dietary intake among older adults with a time perspective that is unique, and results from this study could be used as a basis for prevention strategies encouraging life style changes and disease preventive advice and guidelines for older adults. The increased intake of healthful foods, such as fish, nuts and seeds, fruits and vegetables, is encouraging, but compliance to dietary guidelines could still be improved, and the trend towards an increased alcohol intake among older adults should be paid attention to. The increase in important nutrients related to ageing, such as vitamin D and protein, is positive. However, more than 50% of the participants did not reach the recommended protein intake of 1.2 g/kg BW/day, and about 50% of the women and 30% of the men did not reach the average requirement level of 7.5 μg/day intake of vitamin D, and even less reached the recommended intake (10 μg/day) [8], in the 2014–16 examination.

## Conclusion

This study provides knowledge about dietary intake in relation to nutrient needs and dietary recommendations for older populations. It shows that dietary patterns have changed among 70-year-olds during the past five decades, with an increase in healthful foods and a higher nutrient density in later born birth cohorts. However, the intake of alcohol increased, especially among women. This knowledge contributes to insights in potential risk- and protective factors associated with diet and healthy ageing, and can be useful as a basis for dietary guidelines and used for prevention strategies involving older adults in population-based and health care settings.

## Supplementary information

**Additional file 1.** Food groups.

**Additional file 2.** Check-list STROBE-Nut.

## Data Availability

The datasets used and/or analysed during the current study are available from the corresponding author on reasonable request.

## Abbreviations

AR: Average requirement
BMI: Body mass index
BMR: Basal metabolic rate
BW: Body weight
DH: Diet history
E%: Percentages of energy
EI: Energy intake
Kcal: Kilocalorie
MJ: Megajoul
NFA: National Food Agency
NNR 2012: Nordic Nutrition Recommendations 2012
PAL: Physical activity level
PPSW: The Population Study of Women in Gothenburg
RI: Recommended intake
