# Supplementary material for: Time trends in nutrient intake and dietary patterns among five birth cohorts of 70-year-olds examined 1971–2016: results from the Gothenburg H70 birth cohort studies, Sweden
Source: Nutr J. 2019 Nov 6;18:66. doi: 10.1186/s12937-019-0493-8 (PMC6836447; doi:10.1186/s12937-019-0493-8)
Supplement: Supplementary file 1 — Additional file 1. Food groups. [file 12937_2019_493_MOESM1_ESM.docx]

**Additional file 2.** **Food groups**

| 1 | Fish and shellfish/seafood | Fish and seafood and dishes, caviar and spawn |
| --- | --- | --- |
| 2 | Meat and processed meat | Meat, processed meat, tripe, offal and blood meals and dishes |
| 3 | Poultry | Poultry and poultry dishes |
| 4 | Eggs | Egg and egg dishes |
| 5 | Potatoes | Potatoes and dishes |
| 6 | Vegetables and pulses | Vegetables, root vegetables, mushrooms, pulses and dishes |
| 7 | Fruits and berries | Fruits and berries including canned or dried |
| 8 | Keyhole* milk products | Keyhole milk products; fat content ≤0.7 % and for flavoured products limit for sugars ≤9% |
| 9 | Non-Keyhole milk products | Non-Keyhole milk products, including sweet milk drinks |
| 10 | Cream and crème fraîche | Cream, sour cream and crème fraîche |
| 11 | Cheese | Cheese and cheese dishes |
| 12 | Fast food | Burgers, doner-kebab, falafel, pizza, hot dogs, taco meals |
| 13 | Pasta, rice and food grain | Pasta, rice, food grains and dishes |
| 14 | Bread refined | Bread refined, soft bread and crisp bread <5 % fibre |
| 15 | Bread fibre-rich | Bread fibre-rich, soft bread and crisp bread >5 % fibre |
| 16 | Cereals | Breakfast cereals (hot and cold), porridge and gruel, starch oatmeal |
| 17 | Savoury bakery | Savoury bakery, pasty, savoury pancakes and crêpes, sandwich layer cake, taco shells, crackers, savoury pie |
| 18 | Sweet bakery | Buns, cookies, cakes |
| 19 | Desserts | Sweet pie, crumble, chocolate mousse, cheesecake, sweet soups, ice cream, etc. |
| 20 | Sweet condiments | Sugar, syrup, honey and sweeteners, jam, marmalade, sweet cacao powder |
| 21 | Sweets, candy and chocolate | Sweets, candy, candy bars, chocolate |
| 22 | Salads | Greek salad, chicken salad, pasta salads, etc. |
| 23 | Soups | Soups, broth |
| 24 | Sauces, dressings and condiment | Sauces, dressings, aioli, coleslaw, mayonnaise salads, ketchup, HP sauce |
| 25 | Substitute products | Soya milk, oat milk, coconut milk, tofu, Quorn and soya products |
| 26 | Margarine | Table margarine and soft margarine for cooking, including butter-based |
| 27 | Butter | Butter, lard |
| 28 | Vegetable oil | Vegetable oils |
| 29 | Snacks | Crisps, popcorn, cheese doodles, tortilla chips, etc. |
| 30 | Nuts and seeds | Nuts and seeds including coconut flakes, tahini, peanut butter, etc. |
| 31 | Juice | Fruit and vegetable juices including pure fruit shots |
| 32 | Coffee | Coffee |
| 33 | Tea | Tea |
| 34 | Soda | Soda, lemonade, sports and energy drinks, non-alcoholic cider, regular and light |
| 35 | Alcoholic beverages | Wine, beer, alcoholic cider, drinks, spirits, liqueur ≥1 % alcohol |

*Keyhole is the Swedish National Food Agency-labelling scheme, which guides healthy food choices. For milk and yogurt to meet the criteria for the Keyhole, fat content has to be limited to a maximum of 0·7%, and for flavoured products there is an additional limit for sugars: a maximum of 9 %.
