# Supplementary material for: Time trends in nutrient intake and dietary patterns among five birth cohorts of 70-year-olds examined 1971–2016: results from the Gothenburg H70 birth cohort studies, Sweden
Source: Nutr J. 2019 Nov 6;18:66. doi: 10.1186/s12937-019-0493-8 (PMC6836447; doi:10.1186/s12937-019-0493-8)
Supplement: Supplementary file 2 — Additional file 2. Check-list STROBE-Nut. [file 12937_2019_493_MOESM2_ESM.docx]

**Additional file 3. Check-list STROBE-Nut**

**Title and abstract**

1. The nutritional assessment method is stated in the abstract and as a key word, however not in the title.

**Introduction**

**Background rationale**

1. The scientific background and rationale is described on pages 3-4.

**Objectives**

1. Objectives are described on page 4.

**Methods**

**Study design**

1. Key elements of the study design are described on pages 4-6.

**Settings**

1. Settings, locations and relevant dates are described on pages 4-6.

**Participants**

1. a) Serial cross-sectional design. Selection of participants are described on pages 6-8.

**Variables**

1. 1) Food groups and nutrients are described on pages 9-10.

**Data sources and measurements**

1. 1) Dietary assessment method is described on pages 8-9.
2. 2) Food composition and consumption data, page 8-10.
3. 3) Dietary guidelines are described on pages 9-10.
4. 6) Validity is described on pages 8-9.

**Bias**

1. Pages 9 and 11.

**Study size**

1. Pages 6-8.

**Quantitative variables**

1. Categorisation of food groups on page 10 and in additional file 2. Handling of non-consumers on page 11.

**Statistical method**

1. Statistical analyses are described on page 10-12.

**Results**

**Participants**

1. Missing data is presented in either tables or in table legends and in figure 1.

**Descriptive data**

1. Characteristics of study participants are described on pages 12. Food consumption of the total population and consumers are reported in results and table 5 and 6.

**Outcome data**

1. -

**Main results**

1. Page 12-16. Dietary supplements are not included in the study, page 10.

**Other analyses**

1. Sensitivity analyses. Page 13.

**Discussion**

**Key results**

1. Summarise key results. Page 16.

**Limitations**

1. Pages 20-22.

**Interpretation**

1. Pages 16-20.

**Generalizability**

1. Page 22.
